# Metabolic Phenotypes and Chronic Kidney Disease: A Cross-Sectional Assessment of Patients from a Large Federally Qualified Health Center

**DOI:** 10.3390/life11020175

**Published:** 2021-02-23

**Authors:** Kathleen E. Adair, Nicholas von Waaden, Matthew Rafalski, Burritt W. Hess, Sally P. Weaver, Rodney G. Bowden

**Affiliations:** 1 Department of Health, Human Performance, and Recreation, Baylor University, Waco, TX 76798, USA; nick_von_waaden@baylor.edu; 2 Family Health Center, Waco, TX 76707, USA; mrafalski@wacofhc.org (M.R.); bhess@wacofhc.org (B.W.H.); sweaver@wacofhc.org (S.P.W.); 3 Robbins College of Health and Human Sciences, Baylor University, Waco, TX 76798, USA

**Keywords:** metabolic syndrome, metabolic phenotypes, obesity, chronic kidney disease, renal disease

## Abstract

The purpose of this study is to determine if renal function varies by metabolic phenotype. A total of 9599 patients from a large Federally Qualified Health Center (FQHC) were included in the analysis. Metabolic health was classified as the absence of metabolic abnormalities defined by the National Cholesterol Education Program Adult Treatment Panel III criteria, excluding waist circumference. Obesity was defined as body mass index >30 kg/m^2^ and renal health as an estimated glomerular filtration rate (eGFR) >60 mL/min/1.73 m^2^. Linear and logistic regressions were used to analyze the data. The metabolically healthy overweight (MHO) phenotype had the highest eGFR (104.86 ± 28.76 mL/min/1.72 m^2^) and lowest unadjusted odds of chronic kidney disease (CKD) (OR = 0.46, 95%CI = 0.168, 1.267, *p* = 0.133), while the metabolically unhealthy normal weight (MUN) phenotype demonstrated the lowest eGFR (91.34 ± 33.28 mL/min/1.72 m^2^) and the highest unadjusted odds of CKD (OR = 3.63, *p* < 0.0001). After controlling for age, sex, and smoking status, the metabolically unhealthy obese (MUO) (OR = 1.80, 95%CI = 1.08, 3.00, *p* = 0.024) was the only phenotype with significantly higher odds of CKD as compared to the reference. We demonstrate that the metabolically unhealthy phenotypes have the highest odds of CKD compared to metabolically healthy individuals.

## 1. Introduction

Chronic kidney disease (CKD) is one of the top ten causes of death in the United States (USA), with its prevalence growing from 11.8% in the early 1990s [1] to over 15% in 2019 [2]. Concomitant increases have been observed in obesity and metabolic syndrome over the past three decades, with the prevalence of obesity rising from 22.9% to 42.4% [3] and diagnoses of metabolic syndrome increasing from 25.3% to 34.7% [4]. This milieu is prime for the development and progression of chronic diseases, such as cardiovascular disease (CVD) and CKD, as well as mortality.

Recent research studies have analyzed the impact of metabolic phenotypes, which integrate metabolic risk factors and obesity, in the assessment and prediction of CVD [5,6] and CKD [7]. The metabolic phenotypes consist of four categories: the metabolically healthy normal-weight (MHN), the metabolically healthy obese (MHO), the metabolically unhealthy normal-weight (MUN), and the metabolically unhealthy obese (MUO). The definition of metabolic health has been inconsistent in prior research, but recent studies by Lavie et al. [8] and Kouvari et al. [5] suggested the use of a “strict” definition for metabolic health, which requires the absence of all metabolic risk factors, with the exclusion of obesity. By this definition, individuals with one or more metabolic risk factors (excluding obesity) are considered to be unhealthy.

The strictly defined MHO and MUN are “intriguing” metabolic phenotypes [7], which require further study. Former analyses [5,6] have focused on the prevalence and risk of CVD in the intriguing phenotypes, but no analyses to date have utilized the strict definition of metabolic health to assess renal function and the prevalence of CKD in the MHO and MUN phenotypes. The primary aim of the present study is to analyze the average estimated glomerular filtration rate (eGFR) and the prevalence of CKD in the intriguing metabolic phenotypes (MHO and MUN) utilizing data from a large Federally Qualified Health Center (FQHC) in the South-Central United States. The secondary purpose is to report the prevalence of the strictly defined metabolic phenotypes at a FQHC in the South-Central United States that serves county residents at 200% of the poverty level and below. We hypothesize that the prevalence of CKD will be highest in the metabolically unhealthy normal-weight (MUN) phenotype and lowest in the metabolically healthy obese (MHO) as compared to the reference group (MHN).

## 2. Materials and Methods

The proposed project was approved by the Baylor University Institutional Review Board (IRB) under an expedited review (IRB reference #1465679). Data were acquired from the Epic medical record system by a team of physicians at the FQHC before analysis. All data access and statistical analyses took place at the FQHC site on a locked computer without Internet access. All reports in the present study were drafted on-site at the central FQHC clinic, and data were deidentified and aggregated before leaving the premises so as to keep individual health information protected.

### 2.1. Study Sample

Subjects were not required to sign an informed consent due to the retrospective nature of the study. A query was run by the FQHC physicians to identify active patients with medical health data who were at least 18 years of age and under 89 years of age. The upper age limit was chosen because individuals 90 years and older are few in number and more easily identifiable in the dataset, and, therefore, they were excluded from the analysis to protect health information. An initial cross-sectional dataset identified 16,708 eligible subjects who received medical care at the FQHC and met the age criteria. A subsequent analysis of the data excluded all individuals who were lacking information pertaining to the metabolic phenotypes (body mass index, triglycerides, high-density lipoprotein, diabetes status, and blood pressure) and/or kidney function (serum creatinine), resulting in a final sample of 9599 individuals. Medical charts were used to classify demographic variables, such as race, ethnicity, and smoking status.

### 2.2. Definition of Metabolic Risk Factors

Metabolic risk factors are defined using the criteria outlined in the 2005 revision of the National Cholesterol Education Program (NCEP) Adult Treatment Panel III (ATP III), with the exception of obesity (see Table 1). Waist circumference data were missing in a majority of the subject medical records, and, therefore, the obesity variable was classified using body mass index (BMI). The BMI was calculated using weight (kg) divided by height squared (m^2^). Individuals were considered obese at a BMI ≥ 30 kg/m^2^. Upon clinical diagnosis of a metabolic risk factor or a chronic disease, the FQHC’s medical reporting system categorizes patients into patient registries. The patient registries that were used in our analyses included “Chronic Kidney Disease”, “Diabetes”, “Hypertension”, and “Obstetric”. The registries were used as a secondary determinant in our analyses in cases of missingness or extreme outliers. Due to the rarity of fasting blood glucose samples reported in clinical practice, the classification of hyperglycemia was only determined using the “Diabetes” registry tag in the database. For all other criteria, the most recent laboratory values were used. Subjects were considered to have hypertriglyceridemia if triglyceride levels were ≥150 mg/dL. A measure of high-density lipoprotein (HDL) was used to classify females <50 mg/dL and males <40 mg/dL with dyslipidemia. Subjects were considered hypertensive if they had a resting systolic blood pressure >130 mmHg and/or a resting diastolic blood pressure >85 mmHg, or if they were registered in the “Hypertension” (HTN) registry, which would indicate that HTN was previously diagnosed and/or the subject was prescribed HTN medication.

### 2.3. Definition of Metabolic Phenotypes

The metabolic phenotypes were classified using the strict criteria for metabolic health outlined in recent reports [5,6,8]. These criteria require that metabolic health be defined as the absence of all metabolic risk factors, with the exception of obesity. Obesity has been defined as a BMI ≥ 30 kg/m^2^. Therefore, the metabolically healthy normal weight (MHN) phenotype was defined as having no metabolic risk factors and a BMI < 30 kg/m^2^. The metabolically healthy obese (MHO) phenotype was defined as having no metabolic risk factors and a BMI ≥ 30 kg/m^2^. The metabolically unhealthy normal (MHN) phenotype was defined as having one or more metabolic risk factors and a BMI < 30 kg/m^2^. Lastly, the metabolically unhealthy obese (MUO) phenotype was defined as having one or more metabolic risk factors and a BMI ≥ 30 kg/m^2^.

### 2.4. Renal Outcome Measures

Kidney function was measured using the Modification of Diet in Renal Disease (MDRD) equation [9] which takes into account serum creatinine (SCr), age, sex, and race. The estimated glomerular filtration rate (eGFR) was calculated for each individual utilizing the following equation in a DATA step in the SAS program:

$$\mathrm{eGFR}=175\left({\mathrm{SCr}}^{-1.154}\right)\times \left({\mathrm{age}}^{-0.203}\right)\times \left(0.742\;\mathrm{if}\;\mathrm{female}\right)\times \left(1.212\;\mathrm{if}\;\mathrm{African}\;\mathrm{American}\right)$$

Individuals in the study were considered to have CKD if they had an eGFR < 60 mL/min/1.73 m^2^ or if they were enrolled in the “Chronic Kidney Disease” registry at the FQHC.

### 2.5. Statistical Analysis

The statistical analyses were conducted in SAS version 9.4 (Cary, NC, USA). Descriptive statistics were reported for demographic information. In the case of continuous variables, means and standard deviations were determined using descriptive statistics and analysis of variance (ANOVA) was used to determine if differences existed between means of three or more independent groups. In the case of categorical variables, frequencies or percentages and standard errors were obtained, and χ^2^ tests were conducted to test the difference between groups. Independent and paired samples *t*-tests were used to compare two continuous variables. Correlations were assessed using Pearson’s Correlation Coefficient, *r,* in the case of parametric data, and Spearman’s *ρ* in the case of non-parametric data. Bivariate and multiple linear regression models were used to assess continuous outcome variables, whereas logistic regression was used to assess dichotomous outcome variables. A complete case analysis was conducted. Therefore, missing data were handled by listwise deletion. For all analyses, the level of significance (α-level) was set at 0.05.

## 3. Results

Of the 16,708 eligible subjects, 9599 with complete study information were analyzed. The demographic data for the total group and the four metabolic phenotypes are shown in Table 2. The strictly defined metabolically healthy phenotypes made up 6.41% of the population, and 39.76% of the population was of normal weight (BMI < 30 kg/m^2^). The metabolically healthy obese (MHO) phenotype accounted for the smallest proportion of the population (2.46%), whereas the metabolically unhealthy obese (MUO) accounted for the largest proportion of the population (57.79%). The MHO phenotype had the lowest mean age (41.54 ± 13.56) and the highest proportion of females (78.39%), whereas the MUN phenotype had the highest mean age (57.43 ± 14.29) and the lowest proportion of females (57.29%). The distribution of African Americans was highest in the MUO phenotype, and the distribution of Asian Americans was highest in the MUN phenotype. The normal-weight phenotypes had the highest prevalence of current smokers, whereas the metabolically unhealthy phenotypes had the highest rates of former smokers. Diabetes was identified in 4093 individuals, or 42.64% of the population, and the average hemoglobin A1C (HbA1C) value was 7.05%. The prevalence of coronary artery disease was 9.15%, the prevalence of coronary heart failure was 3.68% in the total population, and cardiovascular disease risk was 15.70%.

Chronic kidney disease was identified in 1166 subjects (12.15%), and the average estimated glomerular filtration rate (eGFR) was 93.69 ± 32.18 mL/min/1.73 m^2^ across the entire sample. The MHO phenotype had a higher average eGFR than any other phenotype at 104.86 ± 26.37 mL/min/1.73 m^2^, whereas the MUN phenotype had the lowest average eGFR at 91.34 ± 33.28 mL/min/1.73 m^2^.

The linear regression models in Table 3 demonstrate unstandardized slope coefficients as mL/min/1.73 m^2^. The unadjusted results revealed a significantly lower eGFR in the MUN and MUO groups (*B* = −10.20, *p* < 0.0001 and *B* = −7.40, *p* < 0.0001, respectively) as compared to the reference group, MHN. This relationship was attenuated after adjusting for mean age in model 2, and this result persisted in models 3 and 4. The logistic regression models in Table 4 demonstrated the odds of CKD for each phenotype. Similar to the linear models, the MUN and MUO phenotypes had greater unadjusted odds (OR = 3.634, *p* < 0.0001 and OR = 2.792, *p* < 0.0001, respectively) of CKD as compared to the referent group. These results were attenuated in the MUN phenotype after adjusting for age, and the attenuation persisted in further adjustments. However, the MUO phenotype maintained higher odds of CKD after adjustment for age, sex, and smoking status, with 1.798 times higher odds of CKD (*p* < 0.0001) in the most adjusted model (Model 4).

## 4. Discussion

### 4.1. Metabolic Phenotypes and CKD

In the observed population, the estimated glomerular filtration rate was highest in the MHO, and the prevalence of CKD was lowest, whereas the MUN phenotype demonstrated the lowest eGFR and highest rate of CKD. Findings in previous literature have been inconsistent, with the highest CKD incidence rates typically found in the MUO phenotype [10,11,12,13,14]. Our findings indicated that the MUO phenotype had the greatest odds of CKD after adjustment for age, sex, and smoking status. Prior longitudinal studies in Iranian and Chinese populations have demonstrated that the MUN [15] and MHO [16] phenotypes, respectively, may have a greater risk of developing CKD over time. However, the cross-sectional nature of our study precludes us from long-term risk-assessment.

The MHO phenotype exhibited better renal health in the unadjusted models. This finding is uncommon, with few studies [17,18] demonstrating higher renal function in obese as compared to normal weight individuals. One study by Hashimoto et al. [11] found no difference in the incidence rates of CKD between the MHN and MHO phenotypes, and other studies have found a lower eGFR and higher rates of CKD in the MHO phenotype as compared to metabolically healthy normal weight individuals [16,19,20,21,22,23]. The higher eGFR in the MHO phenotype found in our study could be due to the cross-sectional nature of the study, which may reflect transient compensation by the kidneys to maintain sodium balance despite higher tubular reabsorption in the state of obesity. The detrimental effects of the obese state on renal function can be seen in longitudinal studies. Bradshaw et al. [24] found a 4-times higher likelihood of the MHO phenotype developing metabolic syndrome over a 9-year follow-up as compared to the MHN phenotype, and Kouvari et al. [5] found that over half of MHO individuals transition to MUO over a 10-year follow-up.

The MUN phenotype, which comprised over one-third of the subjects in our study, demonstrated the lowest eGFR and highest unadjusted odds of CKD. This phenotype often has a more adverse metabolic profile than the MHO. The MUN phenotype has been found to be at greater risk of cardiovascular events [5], mortality [25], and has been correlated with older age, female sex, African American race [24], decreased physical activity, and higher waist circumference [26,27]. However, the MUN phenotype is not typically perceived as high risk [7], and, therefore, it is important to screen the MUN phenotype early for signs of renal dysfunction to ameliorate renal decline before it progresses to CKD. Further study of the strictly defined MUN phenotype may provide more insight into a specific component of the metabolic syndrome that is most significant in the prediction of CKD.

### 4.2. Prevalence of Metabolic Phenotypes

The proportion of metabolically unhealthy and obese phenotypes was high in the observed population, whereas metabolically healthy and normal weight individuals accounted for a small proportion. The metabolically unhealthy obese made up 57.79% of the sample population at the FQHC, which is two-to-three times higher than the frequencies reported in other studies [5,24,25,26,28]. The prevalence of MUO individuals can be partially explained by the largely underserved population at the FQHC as well as its location in the South-Central United States. Using a less strict definition of metabolic health which allowed for up to 2 metabolic abnormalities, Aung et al. [25] reported only 12.92% of a proximate population (San Antonio, TX, USA) as MUO. Using the same strict definition of metabolic health, Kouvari et al. reported 22.49% of the population to be MUO in a homogenous sample of individuals from Greece.

The frequencies of the intriguing metabolic phenotypes (MHO and MUN) in our study were in accordance with previous findings in the literature. The percentage of the MHO phenotype is reported to vary from 6% to 38.4% in prior studies [29] depending on the population and the criteria used to define metabolic health and obesity status. Studies by Pajunen et al. [28] and Kouvari et al. [5], which both used the strict definition of metabolic health, demonstrated a low prevalence of MHO ranging from 3.3% to 5.7%, respectively. Our findings established a similar prevalence at 3.95%, perpetuating the paucity of the strictly defined MHO phenotype. The prevalence of the MUN phenotype in our sample was 35.81%, which also aligns with the findings of Pajunen et al. [28] and Kouvari et al. [5] at 35.7% and 35.56%, respectively. Other studies [24,25,26] have found higher proportions of MHO and lower proportions of MUN, but this is largely attributable to the definitions of metabolic health, which allow for one, two, or three metabolic abnormalities to be considered healthy. Future researchers should consider utilizing a consistent definition for metabolic health to improve comparisons across studies and advance the capacity to predict and prevent chronic diseases.

### 4.3. Limitations

The present study was limited by the cross-sectional nature of the dataset, which prevents further understanding of the causes of CKD or the long-term effects of the four metabolic phenotypes on kidney function. Additionally, the nature of the dataset required that diabetes was classified using the “diabetes” tag in the registry rather than fasting blood glucose measurements. Furthermore, two of the groups, the MHN (n = 379) and MHO (n = 236), were significantly smaller than the MUN and MUO, resulting in difficult comparisons, low statistical power in several analyses, and insufficient evidence to make definitive conclusions about these groups. The scarcity of metabolically healthy individuals could be partially explained by the fact that patients visiting the FQHC are classified as low-socioeconomic status and come to the FQHC seeking medical attention due to lack of insurance and/or funding. Therefore, the subjects in the study were more likely to be unhealthy as compared to other individuals in the surrounding areas. Additionally, we only included individuals who had data that were pertinent to the study, meaning that every subject in the study had been ordered by a physician to have labs drawn. While the FQHC attempts to apply similar care standards for all patients, it is reasonable to assume that healthier patients may not have been sent by physicians for lab work as frequently. The use of body mass index (BMI) to classify obesity status was not ideal. Body mass index alone does not give an indication of central adiposity. In the case of the MUN phenotype, those with higher central or visceral adiposity had the worst long-term survival rates in a previous study [30]. Additionally, it is possible that the small subset of the MHO-classified individuals in our study carried excess weight as muscle or subcutaneous adipose tissue rather than visceral adipose. A measure of waist circumference or a dual-energy x-ray absorptiometry (DEXA) scan would be more appropriate for determining body composition and weight distribution, but is not normally collected in clinical practice. Finally, the high proportion of metabolically unhealthy individuals is due, in part, to the strict definition of metabolic health, which required the absence of all metabolic abnormalities with the exception of obesity.

## 5. Conclusions

To our knowledge, this is the first study to assess the strictly defined metabolic phenotypes and their influence on renal outcomes. While we were limited by the cross-sectional nature of this study, our analysis contributes to a better understanding of the influence of metabolic health and obesity on CKD. We demonstrated that the existence of at least one metabolic abnormality is associated with a lower estimated glomerular filtration rate. Chronic kidney disease was present in a larger percentage of the MUN phenotype, and we found no significant difference between the MHO and the reference group, confirming our hypotheses. In conclusion, the increased risk of CKD in individuals with as little as one metabolic abnormality is indicative that further study of the strictly defined metabolic phenotypes and their influence on renal health is required.

## Figures and Tables

**Table 1:** Criteria for metabolic phenotypes, metabolic abnormalities, and chronic kidney disease.

Category	Criteria	Cut-Off Values for Each Criterion
Metabolic Risk Factors	Obesity	BMI ≥ 30 kg/m^2^
	Hyperglycemia	“Diabetes” tag in FQHC Registry
	Dyslipidemia	TG ≥ 150 mg/dL
	Dyslipidemia (2nd criteria)	HDL-C: <40 mg/dL (M), <50 mg/dL (F)
	Hypertension	SBP > 130 mmHg or DBP > 85 mmHg or Registry
Metabolic Phenotypes	MHN	BMI < 30 and 0 metabolic risk factors
	MHO	BMI ≥ 30 and 0 metabolic risk factors
	MUN	BMI < 30 and ≥1 metabolic risk factors
	MUO	BMI ≥ 30 and ≥1 metabolic risk factors
CKD	Normal or high (G1)	≥90 mL/min/1.73 m^2^
	Mildly decreased (G2)	60–89 mL/min/1.73 m^2^
	Mildly to moderately decreased (G3a)	45–59 mL/min/1.73 m^2^
	Moderately to severely decreased (G3b)	30–44 mL/min/1.73 m^2^
	Severely decreased (G4)	15–29 mL/min/1.73 m^2^
	Kidney failure (G5)	<15 mL/min/1.73 m^2^

Abbreviations: BMI, body mass index; FQHC, Federally Qualified Health Center; TG, triglycerides; HDL-C, high-density lipoprotein-cholesterol; M, males; F, females; SBP, systolic blood pressure; DBP, diastolic blood pressure; MHN, metabolically health normal weight; MHO, metabolically healthy obese; MUN, metabolically unhealthy normal weight; MUO, metabolically unhealthy obese; CKD, chronic kidney disease; G1-5, Assign estimated glomerular filtration rate categories.

**Table 2:** Demographic information for the total sample and metabolic phenotypes.

	Total	MHN	MHO	MUN	MUO	*p*-Value
	(n = 9599)	379 (3.95%)	236 (2.46%)	3437 (35.81%)	5547 (57.79%)	
Age (years)	53.53 (14.32)	44.89 (14.41)	41.54 (13.56)	57.43 (14.29)	52.22 (13.56)	<0.001
BMI (kg/m^2^)	33.06 (8.46)	24.69 (3.42)	36.05 (6.07)	25.81 (3.07)	37.99 (7.33)	<0.001
Sex n, (%)						
Male	3491 (36.37)	123 (32.45)	51 (21.61)	1468 (42.71)	1849 (33.33)	<0.001
Female	6108 (63.63)	256 (67.55)	185 (78.39)	1969 (57.29)	3698 (66.67)	
Race n, (%)						
Caucasian	2953 (30.76)	134 (35.36)	64 (27.12)	1087 (31.63)	1668 (30.07)	0.053
Hispanic or Mexican American	4041 (42.20)	164 (43.39)	117 (49.79)	1447 (42.20)	2313 (41.80)	0.106
African American	2427 (25.28)	72 (19.00)	48 (20.34)	811 (23.60)	1496 (26.97)	<0.001
Asian American	90 (0.94)	4 (1.06)	0	63 (1.83)	23 (0.41)	<0.001
Other/Multi-Racial	13 (0.14)	1 (0.26)	0	5 (0.15)	7 (0.13)	0.839
Smoking n, (%)						
Never	5173 (53.89)	207 (54.62)	155 (65.68)	1670 (48.59)	3141 (56.63)	<0.001
Quit	2298 (23.94)	73 (19.26)	49 (20.76)	819 (23.83)	1357 (24.46)	
Current	2128 (22.17)	99 (26.12)	32 (13.56)	948 (27.58)	1049 (18.91)	
Diabetes	4093 (42.64)	0	0	1364 (39.69)	2729 (49.20)	<0.001
HbA1C	7.05 (4.37)	6.23 (8.74)	5.45 (0.60)	7.10 (4.27)	7.12 (4.19)	<0.001
CAD	878 (9.15)	12 (3.17)	3 (1.27)	362 (10.53)	501 (9.03)	<0.001
CHF	353 (3.68)	2 (0.53)	1 (0.42)	120 (3.49)	230 (4.15)	<0.001
Blood Pressure (SBP/DBP)	131/79	114/73	117/75	132/77	133/80	<0.001
Total Cholesterol	180.95 (42.79)	185.88 (34.80)	186.87 (32.25)	181.28 (45.34)	180.15 (42.01)	0.0085
LDL, per 1 mg/dL	100.76 (35.98)	106.12 (31.18)	108.78 (29.51)	99.88 (36.96)	100.58 (35.86)	<0.001
HDL	46.83 (14.24)	61.93 (13.72)	58.16 (10.89)	48.88 (15.73)	44.05 (12.17)	<0.001
Triglycerides	173.03 (131.38)	89.12 (30.27)	99.69 (29.96)	168.34 (131.38)	184.79 (134.96)	<0.001
SCr	0.88 (0.63)	0.77 (0.21)	0.73 (0.18)	0.93 (0.76)	0.87 (0.56)	<0.001
eGFR, mL/min/1.73 m^2^	93.69 (32.18)	101.54 (26.37)	104.86 (28.76)	91.34 (33.28)	94.14 (31.79)	<0.001
CKD n, (%)	1166 (12.15)	17 (4.49)	5 (2.12)	501 (14.58)	643 (11.59)	<0.001
CVD Risk	15.70 (13.90)	4.84 (4.49)	3.69 (3.40)	17.41 (14.56)	15.90 (13.63)	<0.001

Continuous variables are reported as mean +/− SD, categorical variables are reported as frequency (%), *p*-values are reported for differences between metabolic phenotypes. Abbreviations: BMI, body mass index; HbA1C, hemoglobin A1C; CAD, coronary artery disease; CHF, congestive heart failure; SBP, systolic blood pressure; DBP, diastolic blood pressure; LDL, low-density lipoprotein; HDL, high-density lipoprotein; SCr, serum creatinine; eGFR, estimated glomerular filtration rate; CKD, chronic kidney disease; CVD, cardiovascular disease.

**Table 3:** Linear regression analyses assessing the glomerular filtration rate (eGFR).

	Model 1 ^a^	Model 2 ^b^	Model 3 ^c^	Model 4 ^d^
*Coefficient*	*B (SE)*	*B (SE)*	*B (SE)*	*B (SE)*
MHN (Reference)	101.54 ^†^ (1.65)	93.38 ^†^ (1.49)	93.40 ^†^ (1.55)	95.58 ^†^ (1.59)
MHO	3.23 (2.66)	−0.14 (2.40)	−0.13 (2.40)	−0.42 (2.39)
MUN	−10.20 ^†^ (1.74)	2.42 (1.58)	2.42 (1.59)	2.44 (1.58)
MUO	−7.40 ^†^ (1.70)	−0.02 (1.54)	−0.02 (1.54)	−0.17 (1.54)
Age		−1.01 ^†^ (0.02)	−1.01 ^†^ (0.02)	−0.99 ^†^ (0.02)
Female Sex			−0.06 (0.62)	−0.72 (0.62)
Former Smoker				−3.65 ^†^ (0.74)
Current Smoker				−3.56 ^†^ (0.75)
R^2^	0.007	0.195	0.195	0.198
F value, Pr > F	23.50, <0.0001	582.55, <0.0001	465.99, <0.0001	339.13, <0.0001
Sample (n)	9599	9599	9599	9599

Slope coefficients (*B*) are reported as estimated glomerular filtration rate (eGFR) mL/min/1.73 m^2^ and standard error (*SE*). MHN, metabolically healthy normal weight; MHO, metabolically healthy obese; MUN, metabolically unhealthy normal weight; MUO, metabolically unhealthy obese. ^†^ *p* < 0.0001. ^a^ Model 1 consists of unadjusted results (metabolic phenotypes only). ^b^ Model 2 is adjusted for age (centered at the mean to make the intercept more meaningful). ^c^ Model 3 is adjusted for Model 2 adjustments in addition to sex (male is the reference). ^d^ Model 4 is adjusted for Model 3 adjustments in addition to smoking (1st variable is “used to smoke, quit” the other variable is “current smoker”. The reference is “never smoked”).

**Table 4:** Logistic regression analyses assessing chronic kidney disease (CKD).

	Model 1 ^a^	Model 2 ^b^	Model 3 ^c^	Model 4 ^d^
*Coefficient*	*OR (95% Wald CL)*	*OR (95% Wald CL)*	*OR (95% Wald CL)*	*OR (95% Wald CL)*
MHN (Reference)	1.00 ^†^	1.00 ^†^	1.00 ^†^	1.00 ^†^
MHO	0.461 (0.168, 1.267)	0.584 (0.207, 1.644)	0.561 (0.199, 1.577)	0.558 (0.199, 1.568)
MUN	3.634 ^†^ (2.214, 5.964)	1.573 (0.940, 2.631)	1.603 (0.958, 2.682)	1.612 (0.963, 2.698)
MUO	2.792 ^†^ (1.705, 4.573)	1.827 * (1.098, 3.042)	1.807 * (1.086, 3.008)	1.798 * (1.079, 2.996)
Age		1.082 ^†^ (1.076, 1.089)	1.082 ^†^ (1.076, 1.088)	1.081 ^†^ (1.075, 1.087)
Female Sex			1.390 ^†^ (1.209, 1.598)	1.439 ^†^ (1.248, 1.659)
Former Smoker				1.289 * (1.107, 1.501)
Current Smoker				1.018 (0.853, 1.216)
AIC	7102.296	7102.296	7102.296	7102.296
LR Chi-Sq	78.7828	973.5845	995.4835	1006.8432
Sample (n)	9599	9599	9599	9599

Odds of chronic kidney disease (CKD) reported as Odds Ratio (OR) and 95% Wald Confidence Limit (CL). MHN, metabolically healthy normal weight; MHO, metabolically healthy obese; MUN, metabolically unhealthy normal weight; MUO, metabolically unhealthy obese; LR, likelihood ratio. * *p* < 0.05, ^†^ *p* < 0.0001. ^a^ Model 1 consists of unadjusted results (metabolic phenotypes only). ^b^ Model 2 is adjusted for age (centered at the mean to make the intercept more meaningful). ^c^ Model 3 is adjusted for Model 2 adjustments in addition to sex (male is the reference). ^d^ Model 4 is adjusted for Model 3 adjustments in addition to smoking (1st variable is “used to smoke, quit” the other variable is “current smoker”. The reference is “never smoked”).

## Data Availability

Restrictions apply to the availability of these data. Data was obtained from a Federally Qualified Health Center (FHQC) in Waco, TX and are available from the FHQC.
